# Patterned sequence in the transcriptome of vascular plants

**DOI:** 10.1186/1471-2164-8-173

**Published:** 2007-06-15

**Authors:** Charles F Crane

**Affiliations:** 1 Agricultural Research Service, United States Department of Agriculture, and Department of Botany and Plant Pathology, Purdue University, 915 W. State St., West Lafayette, Indiana 47907-2054, USA

## Abstract

**Background:**

Microsatellites (repeated subsequences based on motifs of one to six nucleotides) are widely used as codominant genetic markers because of their frequent polymorphism and relative selective neutrality. Minisatellites are repeats of motifs having seven or more nucleotides. The large number of EST sequences now available in public databases offers an opportunity to compare microsatellite and minisatellite properties and evaluate their evolution over a broad range of plant taxa.

**Results:**

Repeated motifs from one to 250 nucleotides long were identified in 6793306 expressed sequence tags (ESTs) from 88 genera of vascular plants, using a custom data-processing pipeline that allowed limited variation among repeats. The pipeline processed trimmed but otherwise unfiltered sequence and output nonredundant loci of at least 15 nucleotides, with degree of polymorphism and PCR primers wherever possible. Motifs that were an integral multiple of three in length were more abundant and richer in G/C than other motifs. From 80 to 85% of minisatellite motifs represented repeats within proteins, up to the 228-nucleotide repeat of ubiquitin, but not all of these repeats preserved reading frame. The remaining 15 to 20% of minisatellite motifs were associated with transcribed repetitive elements, e.g., retrotransposons. Relative microsatellite motif frequencies did not correlate tightly to phylogenetic relationship. Evolution of increased microsatellite and EST GC content was evident within the grasses. Microsatellites were less frequent in the transcriptome of genera with large genomes, but there was no evidence for greater dilution of the transcriptome with transposable element transcripts in these genera.

**Conclusion:**

The relatively low correlation of microsatellite spectrum to phylogeny suggests that repeat loci evolve more rapidly than the surrounding sequence, although tissue specificity of the different EST libraries is a complicating factor. In-frame motifs are more abundant and higher in GC than frame-shifting motifs, but most EST minisatellite loci appear to represent repeats in translated sequence, regardless of whether reading frame is preserved. Motifs of four to six nucleotides are as polymorphic in EST collections as the commonly used motifs of two and three nucleotides, and they can be exploited as genetic markers with little additional effort.

## Background

Microsatellites have been defined as tandem direct repeats of motifs (subsequences) of one to six bases [1], and minisatellites as tandem direct repeats of longer motifs. Adjacent repeats comprise a microsatellite locus, which may be simple if there is only one motif, or compound, if there are two or more adjacent blocks of repeated motifs separated by less than some maximum gap of nonrepeated sequence [2]. Microsatellites have been classified as perfect, having no variation among the repeated motifs within the locus, or imperfect, with less than some tolerated percentage (e.g., 10%) of deviant nucleotides over the entire locus. The minimum microsatellite locus has spanned 12 to 20 bases in a number of studies [3-7], although polymorphism has been found to be higher in those at least 20 nucleotides long [8].

Microsatellites are frequently polymorphic within populations or species, and therefore have been widely used as markers in genetic mapping, fingerprinting, and population genetics. New alleles are thought to arise facilely by replication slippage or unequal crossing over at meiosis [9], or by gene conversion [10,11]. To some extent, the same usefulness and evolutionary mechanisms apply to minisatellites. Until relatively recently, the software tools for identifying short, tandemly repeated subsequences have been directed at microsatellites [12-15], although one tool, the Tandem Repeats Finder [16], can currently detect repeated subsequences of any motif length less than 2000 nucleotides. Meanwhile, studies of longer repetitive elements have concentrated on sequences of a few hundred to a few thousand base pairs, e.g., transposable elements, that (with the exception of 5S and 18S-28S ribosomal RNA genes) are generally dispersed in the genome and do not form large tandem arrays with varying copy number. Repetitive subsequences between these length classes remain less analyzed.

Collections of ESTs (expressed sequence tags derived by reverse transcription of messenger RNA) have proven valuable as sources of microsatellite markers. In part, this is because there are many distinct EST sequences in databases [17], microsatellites occur preferentially in nonrepetitive DNA [18] (but see [19] for a contrasting view), and because most EST sequence represents expressed genes that occur singly or in small families in the genome. It also accords with the relatively conserved nature of expressed sequence in relation to the chaotic melange of rapidly evolving repetitive sequences that comprise the bulk of plant genomes [20]; therefore, microsatellite markers derived from the ESTs of one species are likely to be useful in related species [3]. The diminished transferability of microsatellite markers among more distantly related species raises the question of the rate at which such sequences and their flanking sequences evolve, and whether the distribution of microsatellite types carries any phylogenetic information.

Before I was aware of the capacity of Tandem Repeats Finder [16] to identify repeats of minisatellite motifs, I wrote a generic repeat finder in C and several Perl scripts to identify distinct microsatellite loci, group sufficiently close loci into compound loci, condense the collection of microsatellite-bearing sequences to a nonredundant EST set by means of phrap [21], identify likely polymorphisms within the collection, and select plausible PCR primers with primer3 [22]. The resulting pipeline processes any FASTA-formatted sequence collection, which can be highly redundant, and outputs a list of relatively nonredundant microsatellite loci with position, motif, degree of observed polymorphism in the collection, and primers. The findings of this pipeline are here reported and statistically characterized for 88 genera of vascular plants, each represented by a collection of at least 3000 EST sequences in GenBank as of 15 October 2005. Also reported are the results of a maximum-likelihood phylogenetic analysis of the motif frequencies, to express the similarity of these motif frequencies among genera and to relate similarity in microsatellite spectrum to more conventional, broadly based measures of their phylogeny. These findings can be used to test the hypotheses that repeated subsequences are subject to natural selection and that their evolution reflects the evolution of their host genomes.

## Results

### Scope

This study used a custom pipeline to identify repeated subsequences from 1 to 250 bases long in 6793306 sequences and 3681160063 nucleotides from 88 genera of vascular plants. The phrap step reduced the total number of nucleotides to 836433533 in the nonredundant set, for a 77.3% reduction of total sequence length. The genera ranged from *Selaginella *to members of the *Poaceae *and *Asteraceae*, and gymnosperms, *Poaceae*, and *Solanaceae *were comparatively well represented, with at least five genera each. The analysis was repeated three times for each genus, allowing for zero, one, or two single-nucleotide mismatches over all copies of the motif in the repeat locus. In this way, the analysis considered perfect microsatellites, and imperfect microsatellites with a 10% tolerance at minimum locus lengths of 15 and 20 nucleotides. Microsatellites are reported as simple, if they occur in isolation, and compound, if they occur within five bases of another microsatellite. All frequencies are reported from nonredundant sequence as recognized by phrap within the pipeline.

### Frequency by motif length

**Figure 1:**
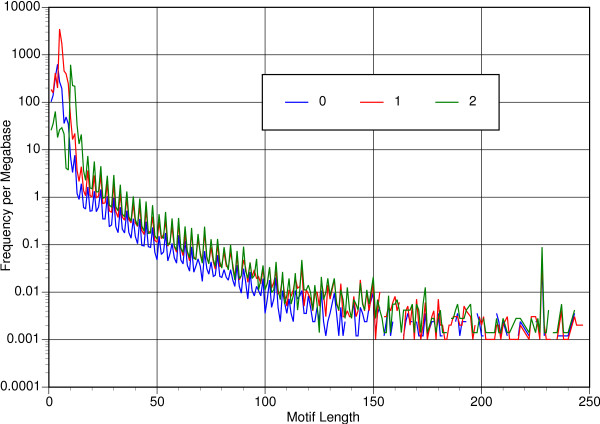
Abundance of microsatellites and minisatellites versus motif length, with zero, one, or two allowed mismatched repeat positions.

Figure 1 shows on a log scale the abundance of microsatellites and minisatellites per megabase in relation to motif length for zero, one, or two allowed mismatched nucleotides. Major shifts in abundance of short motifs reflect the differing threshold values for a minimal microsatellite as the number of allowed mismatches varies. Above decamer motifs there appear periodic peaks wherever the motif length modulus three is zero, i.e., where the minisatellite cannot change the reading frame of the message. This pattern holds even though the abundance of loci with two allowed mismatches falls short of that for zero or one allowed mismatch. The pattern breaks down for motifs longer than 150 bases, largely because the distribution by length becomes discontinuous. These zero values (actually negative infinities) appear at 0.001 in Figure 1. The peak at 228 bases was real and appeared in 13 genera, including dicots, monocots, and a gymnosperm. All 27 instances were related to one another at the peptide level, at a maximum e-value of 7e-21 in a mutual tblastx search. A tblastx of an instance from *Triticum aestivum *revealed no relationship to repetitive elements in RepBase [23], but blastn versus GenBank hit numerous ubiquitin sequences at 1e-69.

The observed motif-length frequencies were compared to those expected from Equations 1 and 2 (given in Materials and Methods) for the pooled sequences of 12 taxonomically divergent genera: *Aquilegia*, *Brassica*, *Citrus*, *Euphorbia*, *Festuca*, *Gossypium*, *Helianthus*, *Ipomoea*, *Lotus*, *Mesembryanthemum*, *Nicotiana*, and *Prunus*. These genera had relatively large EST collections that were still small enough to be processed by phrap without subdivision. As shown in Supplementary Table 1 (see Additional File 1), for most motif lengths the observed repeat frequencies greatly exceeded those expected with random sequence having the same length distribution. Overall, the observed excess was greatest with zero allowed mismatches and least with two. With one allowed mismatch, the observed frequencies of motifs of eight to 11 nucleotides differed relatively little from the frequencies expected from random sequence. Because of the longer minimum locus length with two mismatches, the observed frequency of motifs from 10 through 12 nucleotides was close to the expectation with random sequence. The expected frequencies lacked the observed peaks for motif lengths that were integral multiples of 3 and were many orders of magnitude lower than the observed frequencies for motifs longer than 20 nucleotides.

### Frequency by motif type

Supplementary Table 2 (see Additional File 2) presents per-megabase frequencies of all 501 canonical single-base through six-base motifs in perfect microsatellites for all 88 genera. Its sister tables, Supplementary Tables 3 and 4 (see Additional Files 3 and 4), respectively present the same data for microsatellites with one or two allowed mismatches within the microsatellite locus. These frequencies are itemized for simple, compound, and total loci, and for the fraction of simple loci to simple plus compound loci, as listed in the "Type" column of each table and defined fully in the Abbreviations. The "Type" column of Supplementary Tables 2, 3, and 4 also designates lines for the repeat count and frequency of polymorphism for each canonical motif, itemized for simple loci and submotifs in compound loci, as coded in the Abbreviations. All submotifs within a compound locus were counted as polymorphic if any one of them was, since polymorphism was scored by locus. In Supplementary Tables 2, 3, and 4, the third column is the mean frequency of the respective motif and quantity over all 88 genera. Table 1 combines these mean frequencies over the six length classes to provide a collective view of behavior in regard to motif length.

**Table 1:** Mean microsatellite frequency, repeat count, and polymorphism by motif length

Mismatches	Type	Motif length					
		1	2	3	4	5	6

0	simpfreq	13.574	24.427	50.259	7.826	14.244	17.152
0	compfreq	67.573	114.02	230.762	517.736	203.929	144.361
0	totfreq	81.147	138.447	281.02	525.563	218.174	161.515
0	fracsimp	0.155	0.106	0.153	0.012	0.054	0.099
0	simprepe	18.701	11.593	5.786	4.39	3.227	3.161
0	comprepe	10.287	5.018	3.363	2.038	2.034	2.066
0	simppoly	0.092	0.135	0.167	0.174	0.151	0.185
0	comppoly	0.095	0.112	0.141	0.113	0.121	0.144
1	simpfreq	23.588	27.15	79.092	16.725	33.478	19.782
1	compfreq	132.425	135.595	268.245	146.506	3001.555	1474.271
1	totfreq	156.013	162.745	347.337	163.232	3035.033	1494.054
1	fracsimp	0.141	0.124	0.223	0.1	0.009	0.011
1	simprepe	18.417	10.901	5.759	4.248	3.122	3.147
1	comprepe	12.317	6.526	4.533	3.134	2.007	2.018
1	simppoly	0.102	0.126	0.159	0.165	0.147	0.185
1	comppoly	0.112	0.125	0.157	0.125	0.128	0.138
2	simpfreq	16.906	34.942	45.113	11.936	17.453	20.905
2	compfreq	2.928	5.148	5.326	1.259	1.796	3.169
2	totfreq	19.834	40.091	50.439	13.195	19.249	24.074
2	fracsimp	0.879	0.832	0.903	0.919	0.93	0.879
2	simprepe	24.525	13.555	8.206	5.495	4.297	4.213
2	comprepe	27.141	15.596	8.785	5.695	4.172	3.728
2	simppoly	0.086	0.145	0.169	0.181	0.176	0.205
2	comppoly	0.093	0.167	0.199	0.184	0.191	0.159

Table 1 shows that trinucleotides are the most abundant motif in simple perfect microsatellites, followed consecutively by dinucleotides, hexanucleotides, pentanucleotides, mononucleotides, and tetranucleotides. In contrast, tetranucleotide motifs are the most common in compound perfect microsatellites, followed by trinucleotides, pentanucleotides, hexanucleotides, dinucleotides, and mononucleotides. The total frequency is dominated by the compound microsatellites and follows the same ranking. The fraction of simple to total perfect microsatellites is highest for mononucleotide and trinucleotide motifs, followed by dinucleotides, hexanucleotides, pentanucleotides, and tetranucleotides. The mean repeat count in simple and compound microsatellites was highest for mononucleotide motifs and decreased with increasing motif length, as expected from the minimum length thresholds necessary to complete a locus of at least 15 nucleotides. The mean repeat count was also higher in simple than in compound microsatellites for the same motif, also as expected from the threshold lengths to declare a microsatellite. Hexanucleotide motifs were the most likely to be polymorphic in simple microsatellites; tetranucleotide, trinucleotide, pentanucleotide, dinucleotide, and mononucleotide motifs were consecutively less likely to be polymorphic. In compound microsatellites, polymorphism was most likely in hexanucleotide motifs, then trinucleotide, pentanucleotide, tetranucleotide, dinucleotide, and mononucleotide motifs, and the frequencies were lower than in simple microsatellites. Overall, perfect simple microsatellites were significantly more polymorphic than perfect compound microsatellites by 0.176 to 0.137 (p = 1.03e-13 in a paired t-test).

Increased microsatellite frequencies were expected with one allowed mismatch, because the minimum locus size was 15 nucleotides as in perfect microsatellites. The observed increase ranged from 1.1 to 2.4-fold for simple microsatellites, being greatest for pentanucleotide motifs and least for dinucleotide motifs. For compound microsatellites, the increase was from 1.2 to 14.7-fold, except for a 72% reduction for tetranucleotide motifs, which possibly were being recognized at other motif lengths. Again, the increase was greatest for pentanucleotide motifs. Allowing a mismatch also changed the ranking of frequencies by motif length; with one mismatch, the ranking ran trinucleotides, pentanucleotides, dinucleotides, mononucleotides, hexanucleotides, and tetranucleotides in simple microsatellites, and pentanucleotides, hexanucleotides, trinucleotides, tetranucleotides, dinucleotides, and mononucleotides in compound microsatellites. The frequencies of total microsatellites were ranked in the same order as the frequencies of compound microsatellites. The ratio of simple to total microsatellites increased with one mismatch for dinucleotide, trinucleotide, and tetranucleotide motifs, and decreased with mononucleotide, pentanucleotide, and hexanucleotide motifs. The decrease for hexanucleotides was nearly 90%. The ratio was highest for trinucleotide motifs, then mononucleotide, dinucleotide, tetranucleotide, hexanucleotide, and pentanucleotide motifs. Allowing one mismatch did not greatly change the mean repeat count per locus for simple microsatellites, which again tracked the minimum length criteria for different motif lengths. Allowing a single mismatch did increase the mean repeat count for motifs of four or fewer nucleotides in compound microsatellites. A single mismatch had little effect on the fraction of polymorphic loci, although this fraction increased about 0.015 for motifs of four or fewer nucleotides in compound microsatellites, and trinucleotides replaced hexanucleotides as the class with highest polymorphism in compound microsatellites.

Allowing two mismatches and increasing the minimum locus length from 15 to 20 nucleotides reduced the frequencies of both simple and compound microsatellites up to 150-fold from their values with one mismatch, and reversed their relationship so that simples were 83% (dinucleotides) to 93% (pentanucleotides) of the total. In simple microsatellites, trinucleotide motifs were most abundant, then dinucleotide, hexanucleotide, pentanucleotide, mononucleotide, and tetranucleotide. The ranking was the same for compound and total microsatellites, except that mononucleotide motifs ranked ahead of pentanucleotide motifs. In accordance with the greater length criteria for a minimal microsatellite with two mismatches, the repeat counts were higher for all motif lengths for both simple and compound microsatellites, and fell with increasing motif length. In simple microsatellites, hexanucleotide motifs were the most likely to be polymorphic, then tetranucleotides, pentanucleotides, trinucleotides, dinucleotides, and mononucleotides. In compound loci, the order was trinucleotides, pentanucleotides, tetranucleotides, dinucleotides, hexanucleotides, and mononucleotides. Mononucleotide motifs were least likely to be polymorphic in simple and compound loci over all three numbers of allowed mismatches.

Mean frequencies of eight microsatellite characteristics appear in the third column of Supplementary Tables 2, 3, and 4 (see Additional Files 2, 3, and 4). These frequencies were ranked in descending order within motif length classes to produce Supplementary Tables 5, 6, and 7 (see Additional Files 5, 6, and 7), respectively for zero, one, or two allowed mismatches. Supplementary Tables 8, 9, and 10 (see Additional Files 8, 9, and 10) list in descending order the numbers of genera for which each motif was top ranked, again respectively for zero, one, or two allowed mismatches. Every canonical motif appears in these tables, but 16 hexanucleotide motifs were less than 0.0005 in total frequency with two allowed mismatches: AAACGT, AACGTT, AACTAT, AACTCT, AAGCGT, AAGCTT, AAGTAT, ACCGGT, ACCTAT, ACGCGT, ACGTAG, ACGTAT, AGCGCT, AGGCCT, ATATCG, and ATGCGC.

There was a single clearly most abundant motif for mononucleotides (A), dinucleotides (AG), and trinucleotides (AAG). The most abundant motifs in tetranucleotide, pentanucleotide, and hexanucleotide microsatellites were AAAG, AAAAG, AAAAAG, or motifs with A and two or three Gs, but these were relatively less abundant than the top-ranked loci with shorter motifs. PolyA motifs ending in a single T or C were also abundant overall, but other runs of a single letter were not particularly common. Motif rankings diverged between simple and compound loci with increasing motif length. Simple loci were typically from 5 to 20% of all loci with zero or one mismatch, but at least 80% of loci were simple with two allowed mismatches.

### Correlations among microsatellite properties

Table 2 gives rank correlation coefficients (Spearman's rho, calculated using R [24]) for seven types of comparisons at each mismatch level and motif length from three through six. The comparisons are designated according to the same codes as the "Type" column in Supplementary Table 2. The null hypothesis was no correlation at all. Although R could not calculate exact p-values in the presence of ties in rank, it did produce approximate p-values that were accepted for the determination of significance. These p-values for the combined motif lengths usually tracked the p-values for hexanucleotide motifs, which contributed 350 out of the combined 501 data values. Motif frequency was strongly, positively correlated between compound and simple loci, although the correlation weakened for hexanucleotide motifs. Repeat count was less strongly correlated between compound and simple loci, although the correlation was highly significant for hexanucleotide and collective repeats, and for tetranucleotide motifs with one or two mismatches. Polymorphism was uncorrelated between simple and compound loci with zero or two mismatches; the correlations with one mismatch were low to moderate, but mostly significant. Repeat count was not significantly correlated to frequency of simple loci, except in hexanucleotide motifs and the collective motifs that they dominated. Repeat count was more correlated to frequency for compound loci, where pentanucleotides also reached significant values with one or two mismatches. Polymorphism was significantly correlated to repeat count in simple and compound loci with hexanucleotide motifs and collectively, but not for shorter motifs, and the correlations for trinucleotide motifs were mostly somewhat negative.

**Table 2:** Spearman rank correlation between features in relation to microsatellite motif length

x	y	mismatches	3-mer (8 d.f.)	4-mer (31 d.f.)	5-mer (99 d.f.)	6-mer (348 d.f.)	all (499 d.f.)
compfreq	simpfreq	0	0.915***	0.714***	0.860***	0.789***	0.840***
compfreq	simpfreq	1	0.964***	0.900***	0.901***	0.693***	0.732***
compfreq	simpfreq	2	0.960***	0.809***	0.844***	0.755***	0.805***
simprepe	comprepe	0	0.736*	0.19	0.138	0.390***	0.313***
simprepe	comprepe	1	0.467	0.556***	0.183	0.519***	0.660***
simprepe	comprepe	2	0.758*	0.494**	0.067	0.370***	0.488***
simppoly	comppoly	0	0.024	0.029	0.201*	0.068	0.127**
simppoly	comppoly	1	0.701*	0.452**	0.291**	0.160**	0.222***
simppoly	comppoly	2	0.284	-0.217	0.037	0.143**	0.090*
simpfreq	simprepe	0	0.079	0.327	-0.053	0.486***	0.487***
simpfreq	simprepe	1	0.2	0.073	0.062	0.487***	0.404***
simpfreq	simprepe	2	0.03	0.303	-0.159	0.315***	0.363***
compfreq	comprepe	0	0.517	0.167	0.236*	0.296***	0.100*
compfreq	comprepe	1	0.333	0.015	0.433***	0.204***	-0.05
compfreq	comprepe	2	-0.17	0.432*	0.314**	0.609***	0.601***
simprepe	simppoly	0	-0.018	0.073	0.168	0.185***	0.136**
simprepe	simppoly	1	-0.255	0.263	0.055	0.130*	0.115*
simprepe	simppoly	2	-0.122	0.181	0.087	0.228***	0.129**
comprepe	comppoly	0	-0.25	0.534**	-0.049	-0.029	0.068
comprepe	comppoly	1	0.105	0.298	-0.099	0.201***	0.142**
comprepe	comppoly	2	-0.614	0.082	0.292**	0.434***	0.394***
*, p <= 0.05; **, p <= 0.01; ***, p <= 0.001							

Values of the eight traits defined in the Abbreviations are listed versus motif length and GC content for perfect microsatellites in Table 3, for one mismatch in Table 4, and for two mismatches in Table 5. Monoclinal trends with respect to GC content were uncommon; examples include repeat count in simple trinucleotide loci, which declined with increasing GC over all three mismatch levels, and total frequency in tetranucleotide loci with zero or one mismatches. A simple peak or dip sometimes was observed, e.g., the total frequency of hexanucleotide loci with 2/3 GC was minimal with zero mismatches, and the polymorphism of simple pentanucleotide loci with 3/5 GC was maximal with zero or one mismatch. More generally, there were two peaks or dips in the pattern. The patterns were fitted to a quadratic model of the form y = a + bx + cx^2^, using function glm() in R, to produce Supplementary Table 11 (see Additional File 11). The significance of coefficients in this table is based on t-values, and 151 of the 288 reported coefficients differed significantly from zero at p <= 0.05. There were 54 coefficients that were significant at p <= 0.01, and 50 more that were significant at p <= 0.001. The coefficients were particularly likely to be significant for pentanucleotide motifs with a single mismatch and for intercepts in general. The coefficients of the linear and quadratic terms were more likely to be significant for abundance than for the other traits, and usually the linear coefficient was negative and the quadratic coefficient was positive for abundance. The relative abundance of simple and compound loci did not respond much to GC content, and neither did the repeat count at any number of mismatches. Polymorphism frequently had a significantly negative quadratic coefficient, especially with zero or one mismatch in pentanucleotide and hexanucleotide motifs, meaning that the GC-rich motifs were overall less polymorphic.

**Table 3:** Mean frequency, repeat count, and polymorphism by motif length and GC content in perfect microsatellites

Length	GC content	simpfreq	compfreq	totfreq	fracsimp	simprepe	comprepe	simppoly	comppoly
3	0	3.464	16.996	20.46	0.144	6.254	3.412	0.168	0.115
	0.333	5.178	24.711	29.889	0.142	5.864	3.369	0.166	0.142
	0.667	4.66	21.682	26.342	0.163	5.646	3.354	0.169	0.146
	1	7.442	28.194	35.636	0.167	5.564	3.323	0.158	0.146
4	0	0.8	29.279	30.079	0.022	4.407	2.058	0.131	0.106
	0.25	0.353	24.872	25.225	0.012	4.555	2.042	0.147	0.108
	0.5	0.158	12.031	12.189	0.013	4.317	2.039	0.195	0.121
	0.75	0.148	10.598	10.746	0.012	4.345	2.03	0.169	0.113
	1	0.085	9.502	9.587	0.003	4.373	2.018	0.217	0.091
5	0	0.663	6.274	6.937	0.076	3.171	2.051	0.123	0.103
	0.2	0.245	3.426	3.671	0.05	3.194	2.031	0.144	0.112
	0.4	0.118	2.056	2.174	0.051	3.242	2.03	0.149	0.121
	0.6	0.091	1.293	1.384	0.056	3.264	2.04	0.163	0.135
	0.8	0.083	1.084	1.167	0.055	3.174	2.033	0.146	0.109
	1	0.104	1.927	2.03	0.055	3.18	2.026	0.122	0.099
6	0	0.099	1.074	1.173	0.052	3.093	2.054	0.108	0.109
	0.167	0.048	0.674	0.721	0.046	3.085	2.05	0.162	0.122
	0.333	0.034	0.399	0.433	0.067	3.188	2.046	0.188	0.142
	0.5	0.047	0.39	0.436	0.098	3.202	2.065	0.188	0.147
	0.667	0.05	0.286	0.335	0.133	3.106	2.086	0.192	0.152
	0.833	0.081	0.46	0.541	0.151	3.202	2.092	0.187	0.151
	1	0.084	0.462	0.546	0.137	3.079	2.068	0.183	0.116

**Table 4:** Mean frequency, repeat count, and polymorphism by motif length and GC content in microsatellites with one allowed mismatched repeat position

Length	GC content	simpfreq	compfreq	totfreq	fracsimp	simprepe	comprepe	simppoly	comppoly
3	0	4.578	17.571	22.148	0.197	5.912	4.614	0.151	0.123
	0.333	7.923	27.527	35.45	0.211	5.789	4.508	0.156	0.157
	0.667	7.718	25.59	33.307	0.236	5.705	4.553	0.167	0.166
	1	11.949	38.208	50.157	0.246	5.698	4.472	0.144	0.158
4	0	1.466	11.444	12.91	0.121	4.224	3.156	0.122	0.11
	0.25	0.773	7.478	8.251	0.091	4.277	3.152	0.168	0.127
	0.5	0.366	3.009	3.375	0.105	4.25	3.13	0.19	0.13
	0.75	0.303	2.466	2.768	0.103	4.24	3.136	0.141	0.117
	1	0.217	2.475	2.692	0.067	4.179	3.064	0.13	0.132
5	0	1.364	71.877	73.241	0.016	3.11	2.015	0.117	0.113
	0.2	0.627	47.195	47.822	0.01	3.094	2.009	0.14	0.12
	0.4	0.295	31.143	31.438	0.008	3.123	2.007	0.15	0.129
	0.6	0.201	21.117	21.317	0.008	3.145	2.007	0.158	0.133
	0.8	0.173	17.65	17.823	0.008	3.115	2.007	0.138	0.13
	1	0.248	25.358	25.607	0.007	3.065	2.008	0.12	0.125
6	0	0.019	6.452	6.471	0.003	3.096	2.009	0.141	0.11
	0.167	0.039	6.483	6.523	0.005	3.136	2.01	0.155	0.118
	0.333	0.045	4.697	4.741	0.008	3.121	2.011	0.176	0.13
	0.5	0.061	4.169	4.231	0.012	3.18	2.017	0.194	0.142
	0.667	0.055	2.999	3.054	0.015	3.158	2.025	0.196	0.147
	0.833	0.094	3.778	3.871	0.019	3.199	2.031	0.191	0.146
	1	0.089	3.137	3.226	0.015	2.58	2.019	0.193	0.137

**Table 5:** Mean frequency, repeat count, and polymorphism by motif length and GC content in microsatellites with two allowed mismatched repeat positions

Length	GC content	simpfreq	compfreq	totfreq	fracsimp	simprepe	comprepe	simppoly	comppoly
3	0	3.226	0.447	3.673	0.882	8.739	9.487	0.189	0.145
	0.333	4.822	0.595	5.417	0.905	8.309	9.217	0.163	0.189
	0.667	4.037	0.483	4.521	0.896	8.051	8.418	0.172	0.214
	1	6.45	0.566	7.017	0.942	7.888	7.817	0.162	0.231
4	0	1.107	0.101	1.208	0.917	5.497	5.513	0.133	0.152
	0.25	0.577	0.074	0.651	0.919	5.585	5.878	0.191	0.169
	0.5	0.225	0.018	0.244	0.928	5.475	5.772	0.185	0.206
	0.75	0.237	0.024	0.262	0.909	5.504	5.578	0.174	0.176
	1	0.14	0.015	0.156	0.899	5.224	5.109	0.201	0.166
5	0	0.763	0.073	0.836	0.918	4.188	4.283	0.129	0.144
	0.2	0.314	0.035	0.348	0.925	4.276	4.333	0.176	0.185
	0.4	0.14	0.014	0.154	0.925	4.318	4.507	0.167	0.245
	0.6	0.11	0.009	0.119	0.939	4.341	3.825	0.19	0.142
	0.8	0.109	0.013	0.121	0.939	4.242	3.99	0.178	0.207
	1	0.143	0.022	0.165	0.879	4.12	4.299	0.168	0.137
6	0	0.101	0.016	0.117	0.878	4.213	4.385	0.148	0.165
	0.167	0.06	0.008	0.069	0.867	4.167	3.86	0.168	0.116
	0.333	0.042	0.006	0.048	0.889	4.19	3.392	0.195	0.115
	0.5	0.058	0.009	0.067	0.9	4.323	3.682	0.234	0.164
	0.667	0.056	0.01	0.067	0.839	4.103	3.846	0.206	0.19
	0.833	0.104	0.014	0.118	0.897	4.261	4.23	0.188	0.207
	1	0.102	0.01	0.112	0.916	4.137	3.57	0.143	0.219

The GC content of microsatellites was correlated with GC content of the entire transcriptome of each genus, but the correlation diminished for short motifs with two mismatches, where the recognized microsatellite loci were longer. Over all perfect microsatellites, Pearson's correlation coefficient was 0.960; with one allowed mismatch, it was 0.984; and for two mismatches, it was 0.971. The respective values for loci or subloci based on two- to six-base motifs were 0.953, 0.978, and 0.865, for zero, one, or two allowed mismatches. Microsatellite GC content outpaced increases in transcriptome GC content; the GC content of microsatellites in GC-rich transcriptomes exceeded the transcriptome's GC content, and the GC content of microsatellites in GC-poor transcriptomes fell short of the transcriptome's GC content. For perfect microsatellites, the regression slopes of microsatellite to whole-transcriptomic GC content (y = a + bx by glm in R) were 2.06 for two- through six-base motifs, and 1.96 for all motifs. These slopes respectively became 1.85 and 1.71 for one mismatch, and 2.54 and 1.79 for two mismatches. All of these values differed from zero at p-values less than 1e-16, as did all their intercepts. Fitting a quadratic model (y = a + bx + cx^2^) gave positive intercepts that did not differ significantly from zero, negative b that did not differ significantly from zero, and positive c between 1.72 and 4.54 that was not significant for short motifs with two mismatches, or significant at 0.082 for all motifs in perfect microsatellites, down to 0.005 for short motifs with a single allowed mismatch. The significant values of c were between 1.72 and 2.61.

**Figure 2:**
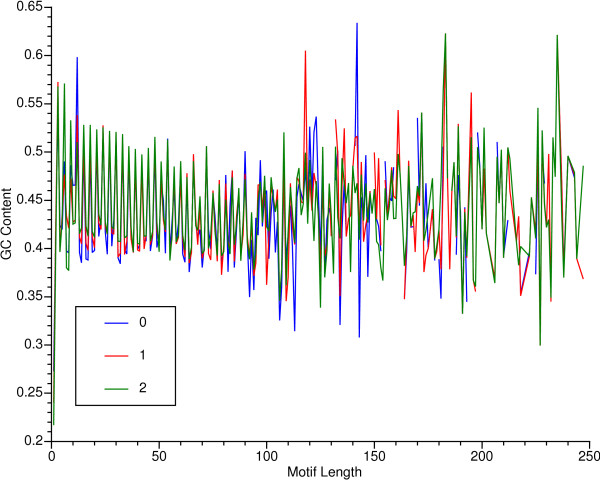
GC content versus motif length for tandemly repeated subsequences. There are separate traces for zero, one, and two allowed mismatched repeat positions.

The GC content of microsatellites and minisatellites did not vary in proportion to motif length between 1 and 250 bases; the regression coefficients for a linear model (y = a + bx) were 6.26e-5, 3.13e-5, and 4.25e-5 for zero, one, or two mismatches, none of which differed significantly from zero. However, the slope between 1 and 90 base pairs was significantly negative at p < 0.0005 for motif lengths integrally divisible by 3 in all three mismatch classes. Also, GC content spiked at integral multiples of three (Fig. 2) for motif lengths up to 90. Motifs that frame-shifted their message had a significantly lower GC content that those that preserved the reading frame, at p-values less than 6e-12 in t-tests, regardless of mismatch level. Motifs that shifted their reading frame by one nucleotide did not differ significantly in GC content from those that shifted their reading frame by two nucleotides.

### Comparative microsatellite frequencies among taxa

Top ranking was well distributed among genera for the 149 dinucleotide through pentanucleotide motifs. For perfect microsatellites, 42 genera ranked first in frequency of at least one motif. For one allowed mismatching nucleotide, 26 genera ranked first, and for two allowed mismatching nucleotides, 50 genera ranked first. In contrast, one genus, *Pisum*, ranked last (0) for 69 of these motifs, in accordance with its smallest nonredundant sample size of 1076 nonredundant sequences.

Similarity was tested by mean Euclidean distance within groups of related genera for total frequency in simple plus compound loci, repeat count in simple loci, and frequency of polymorphism in simple loci. This mean distance was compared to the distribution of 20000 mean distances in groups of the same size for the same character and number of allowed mismatches, drawn at random without replacement from the set of all 88 genera. The distance is based on the 499 canonical dinucleotide through hexanucleotide motifs; A and C were excluded, because either can appear spuriously in low-resolution sequence, and A can represent untrimmed polyA tails, especially any polyA trapped in chimeric clones. The results appear in Tables 6, 7, and 8 for 18 groups ranging in diversity from the grass subfamilies *Pooideae *and *Panicoideae *to the three included non-seed plants. Genera were significantly close together for total frequency in the conifers, *Solanaceae*, and *Asteraceae*, at all three allowed numbers of mismatches. Otherwise, total motif frequencies for genera within the indicated families and orders were not significantly closer than expected by chance. Also, there were no significant differences of observed mean distances from random expectation for repeat count in these three tables. Mean distances for polymorphism were significantly close only for the three lower vascular plants with zero or two mismatches, meaning that their average degree of polymorphism for each motif was significantly similar.

**Table 6:** Mean within-group Euclidean distance of genera for total motif frequency, mean repeat count, and polymorphism in perfect microsatellite loci

Group	Mean Euclidean distance		
	Total frequency	Repeat count	Polymorphism

Lower vascular plants: *Selaginella*, *Ceratopteris*, *Adiantum*	128.957	41.14	1.534**
*Pinaceae*: *Pinus*, *Picea*, *Pseudotsuga*	52.489**	52.622	7.458
*Coniferales*: *Pinus*, *Picea*, *Pseudotsuga*, *Cryptomeria*	53.881****	54.362	6.594
Other gymnosperms: *Cycas*, *Zamia*, *Ginkgo*,*Gnetum*, *Welwitschia*	127.215	33.696	2.375
*Caryophyllales*: *Beta*, *Mesembryanthemum*, *Tamarix*	252.658	44.591	4.749
*Rosaceae*: *Rosa*, *Prunus*, *Malus*, *Fragaria*	106.051	45.096	5.529
*Fabaceae*: *Phaseolus*, *Glycine*, *Lotus*, *Medicago*, *Pisum*	117.741	51.308	6.615
*Malphigiales*: *Euphorbia*, *Manihot*, *Populus*, *Bruguiera*	183.203	53.741	5.709
*Brassicaceae*: *Brassica*, *Arabidopsis*, *Thellungiella*	126.63	47.548	5.882
*Lamiales*: *Sesamum*, *Salvia*, *Antirrhinum*, *Mimulus*, *Triphysaria*	161.912	38.581	3.548
*Solanaceae*: *Solanum*, *Lycopersicon*, *Capsicum*, *Nicotiana*, *Petunia*	62.281***	44.787	6.565
*Asteraceae*: *Helianthus*, *Stevia*,*Cichorium*, *Lactuca*, *Taraxacum*, *Zinnia*, *Gerbera*	87.183**	43.732	4.179
*Poaceae*: *Triticum*, *Aegilops*,*Hordeum*, *Secale*, *Avena*, *Oryza*, *Cynodon*, *Eragrostis*, *Panicum*, *Pennisetum*, *Saccharum*, *Sorghum*, *Zea*, *Lolium*, *Festuca*	248.316	49.556	5.014
*Pooideae*: *Aegilops*, *Triticum*, *Secale*, *Hordeum*, *Avena*, *Festuca*, *Lolium*	179.427	49.285	5.018
*Triticeae*: *Aegilops*, *Triticum*, *Secale*, *Hordeum*	176.441	53.458	6.143
*Panicoideae*: *Panicum*, *Pennisetum*, *Saccharum*, *Sorghum*, *Zea*	205.429	47.349	5.285
*Asparagales*: *Asparagus*, *Allium*, *Lycoris*	160.725	36.687	3.221
Other monocots: *Acorus*, *Zantedeschia*, *Ananas*	185.791	37.821	2.937
Group 1: *Adiantum*, *Ceratopteris*, *Cichorium*, *Welwitschia*	88.991	36.793	1.892**
Group 2: *Cycas*, *Ginkgo*, *Stevia*, *Triphysaria*, *Zea*, *Zinnia*	63.819****	41.682	3.425
Group 3: *Allium*, *Cryptomeria*, *Picea*, *Pinus*, *Zamia*	73.179**	52.924	5.982
Group 4: *Amborella*, *Bruguiera*, *Eschscholzia*, *Mesembryanthemum*	152.077	43.471	3.194
Group 5: *Beta*, *Brassica*, *Liriodendron*, *Malus*,*Rosa*, *Saruma*, *Thellungiella*	85.727**	43.56	4.838
Group 6: *Aquilegia*, *Arabidopsis*, *Cucumis*, *Euphorbia*	113.958	49.053	6.698
Group 7: *Manihot*, *Persea*, *Theobroma*	51.524***	32.473	2.117
Group 8: *Cynodon*, *Gossypium*, *Lycopersicon*, *Medicago*, *Pisum*, *Solanum*	271.98	51.628	6.96
Group 9: *Nuphar*, *Panax*	54.605	37.591	1.207
Group 10: *Glycine*, *Ipomoea*, *Juglans*, *Populus*, *Prunus*, *Vitis*	107.381	48.805	7.114
Group 11: *Helianthus*, *Lactuca*, *Lotus*, *Phaseolus*	69.613**	44.171	6.412
Group 12: *Antirrhinum*, *Capsicum*, *Gerbera*, *Taraxacum*	51.245****	42.076	4.515
Group 13: *Acorus*, *Ananas*, *Asparagus*, *Eucalyptus*, *Sesamum*, *Vaccinium*	104.739	35.615	2.156**
Group 14: *Citrus*, *Lycoris*, *Mimulus*, *Poncirus*, *Tamarix*	134.97	44.042	6.047
Group 15: *Aegilops*, *Hordeum*, *Oryza*, *Panicum*, *Secale*, *Sorghum*, *Triticum*	177.011	49.198	6.187
Group 16: *Avena*, *Eragrostis*, *Festuca*, *Lolium*, *Pennisetum*	95.789	39.884	2.992

**Table 7:** Mean within-group Euclidean distance of genera for total motif frequency, mean repeat count, and polymorphism in microsatellite loci with one allowed mismatch

Group	Mean Euclidean distance		
	Total frequency	Repeat count	Polymorphism

Lower vascular plants: *Selaginella*, *Ceratopteris*, *Adiantum*	229.155	41.982	2.604
*Pinaceae*: *Pinus*, *Picea*, *Pseudotsuga*	139.117	50.474	7.411
*Coniferales*: *Pinus*, *Picea*, *Pseudotsuga*, *Cryptomeria*	131.475**	51.5	6.603
Other gymnosperms: *Cycas*, *Zamia*, *Gnetum*, *Welwitschia*, *Ginkgo*	255.205	35.919	3.384
*Caryophyllales*: *Beta*, *Mesembryanthemum*, *Tamarix*	330.728	44.916	5.173
*Rosaceae*: *Rosa*, *Prunus*, *Malus*, *Fragaria*	181.561	46.964	5.931
*Fabaceae*: *Phaseolus*, *Glycine*, *Lotus*, *Medicago*, *Pisum*	252.623	52.162	6.718
*Malphigiales*: *Euphorbia*, *Manihot*, *Populus*, *Bruguiera*	287.749	48.202	6.216
*Brassicaceae*: *Brassica*, *Arabidopsis*, *Thellungiella*	259.861	48.064	6.67
*Solanaceae*: *Solanum*, *Lycopersicon*, *Capsicum*, *Nicotiana*, *Petunia*	128.443***	45.334	5.81
*Lamiales*: *Sesamum*, *Salvia*, *Antirrhinum*, *Mimulus*, *Triphysaria*	261.175	36.538	3.962
*Asteraceae*: *Helianthus*, *Stevia*, *Cichorium*, *Lactuca*, *Taraxacum*, *Zinnia*, *Gerbera*	167.495**	43.556	4.585
*Poaceae*: *Triticum*, *Hordeum*, *Secale*, *Avena*, *Oryza*, *Cynodon*, *Eragrostis*, *Panicum*, *Pennisetum*, *Saccharum*, *Sorghum*, *Zea*, *Lolium*, *Festuca*, *Aegilops*	403.092	47.223	5.678
*Pooideae*: *Aegilops*, *Triticum*, *Secale*, *Hordeum*, *Avena*, *Festuca*, *Lolium*	302.884	45.347	5.808
*Triticeae*: *Aegilops*, *Triticum*, *Secale*, *Hordeum*	297.79	47.58	6.977
*Panicoideae*: *Panicum*, *Pennisetum*, *Saccharum*, *Sorghum*, *Zea*	303.835	45.552	5.879
*Asparagales*: *Asparagus*, *Allium*, *Lycoris*	270.545	35.907	4.317
Other monocots: *Acorus*, *Zantedeschia*, *Ananas*	218.631	37.964	3.657
Group 1: *Adiantum*, *Avena*, *Ceratopteris*, *Cycas*, *Ginkgo*, *Pseudotsuga*	164.376**	35.474	3.313
Group 2: *Cryptomeria*, *Picea*, *Pinus*, *Tamarix*	165.313	51.348	6.861
Group 3: *Amborella*, *Bruguiera*, *Eschscholzia*, *Mesembryanthemum*	238.256	39.407	3.454
Group 4: *Beta*, *Brassica*, *Ipomoea*, *Juglans*, *Malus*, *Saruma*	145.794**	46.777	6.325
Group 5: *Gnetum*, *Manihot*, *Phaseolus*, *Prunus*	184.201	42.602	5.33
Group 6: *Aquilegia*, *Cucumis*, *Euphorbia*, *Helianthus*, *Lactuca*, *Lotus*, *Lycopersicon*	152.767**	43.374	6.477
Group 7: *Nuphar*, *Panax*, *Persea*, *Theobroma*	114.516***	35.681	3.258
Group 8: *Liriodendron*,*Rosa*, *Sesamum*, *Thellungiella*, *Vaccinium*	138.397**	33.618	2.432***
Group 9: *Arabidopsis*, *Cynodon*, *Glycine*, *Gossypium*, *Medicago*, *Populus*, *Solanum*, *Vitis*	361.635	47.201	7.402
Group 10: *Antirrhinum*, *Cichorium*, *Gerbera*, *Pisum*, *Stevia*, *Taraxacum*, *Triphysaria*, *Zinnia*	141.561****	40.511	3.768
Group 11: *Citrus*, *Lycoris*, *Mimulus*, *Poncirus*, *Zamia*	256.198	43.848	5.768
Group 12: *Aegilops*, *Hordeum*, *Oryza*, *Panicum*, *Saccharum*, *Secale*, *Sorghum*, *Triticum*, *Zea*	278.936	42.717	6.583
Group 13: *Eragrostis*, *Festuca*, *Lolium*, *Pennisetum*	126.825**	42.493	3.569

**Table 8:** Mean within-group Euclidean distance of genera for total motif frequency, mean repeat count, and polymorphism in microsatellite loci with two allowed mismatches

Group	Mean Euclidean distance		
	Total frequency	Repeat count	Polymorphism

Lower vascular plants: *Selaginella*, *Ceratopteris*, *Adiantum*	36.903	56.8	1.411***
*Pinaceae*: *Pinus*, *Picea*, *Pseudotsuga*	10.519****	69.929	7.196
*Coniferales*: *Pinus*, *Picea*, *Pseudotsuga*, *Cryptomeria*	14.546***	67.788	6.506
Other gymnosperms: *Cycas*, *Zamia*, *Gnetum*, *Welwitschia*, *Ginkgo*	33.035	50.695	3.059
*Caryophyllales*: *Beta*, *Mesembryanthemum*, *Tamarix*	67.331	62.875	4.632
*Rosaceae*: *Rosa*, *Prunus*, *Malus*, *Fragaria*	39.626	60.01	5.567
*Fabaceae*: *Phaseolus*, *Glycine*, *Lotus*, *Medicago*, *Pisum*	30.364	66.912	6.765
*Brassicaceae*: *Brassica*, *Arabidopsis*, *Thellungiella*	23.864	59.554	6.267
*Malphigiales*: *Euphorbia*, *Manihot*, *Populus*, *Bruguiera*	88.122	67.092	6.054
*Solanaceae*: *Solanum*, *Lycopersicon*, *Capsicum*, *Nicotiana*, *Petunia*	13.763****	58.708	6.594
*Lamiales*: *Sesamum*, *Salvia*, *Antirrhinum*, *Mimulus*, *Triphysaria*	46.143	48.69	3.589
*Asteraceae*: *Helianthus*, *Stevia*, *Cichorium*, *Lactuca*, *Taraxacum*, *Zinnia*, *Gerbera*	25.452**	57.543	4.426
*Poaceae*: *Triticum*, *Hordeum*, *Secale*, *Avena*, *Oryza*, *Cynodon*, *Eragrostis*, *Panicum*, *Pennisetum*, *Saccharum*, *Sorghum*, *Zea*, *Lolium*, *Festuca*, *Aegilops*	50.746	63.157	5.731
*Pooideae*: *Aegilops*, *Triticum*, *Secale*, *Hordeum*, *Avena*, *Festuca*, *Lolium*	43.303	62.033	5.498
*Triticeae*: *Aegilops*, *Triticum*, *Secale*, *Hordeum*	49.094	65.451	6.753
*Panicoideae*: *Panicum*, *Pennisetum*, *Saccharum*, *Sorghum*, *Zea*	37.194	60.681	6.531
*Asparagales*: *Asparagus*, *Allium*, *Lycoris*	42.527	44.446	3.14
Other monocots: *Acorus*, *Zantedeschia*, *Ananas*	131.502	50.358	3.087
Group 1: *Amborella*, *Bruguiera*, *Eschscholzia*, *Mesembryanthemum*	69.26	54.761	3.502
Group 2: *Juglans*, *Liriodendron*, *Rosa*, *Vaccinium*	23.313	43.599	3.106
Group 3: *Glycine*, *Populus*, *Vitis*	14.049**	57.31	8.335
Group 4: *Brassica*, *Ipomoea*, *Saruma*	15.270**	60.331	6.408
Group 5: *Aquilegia*, *Arabidopsis*, *Cucumis*, *Euphorbia*, *Lactuca*, *Medicago*, *Thellungiella*	31.587	62.644	6.379
Group 6: *Gerbera*, *Nuphar*, *Persea*, *Prunus*, *Salvia*, *Sesamum*	24.786**	51.956	3.878
Group 7: *Citrus*, *Hedyotis*, *Mimulus*, *Poncirus*, *Tamarix*	30.045	59.185	6.25
Group 8: *Solanum*, *Lycopersicon*, *Gossypium*	17.538**	58.194	8.405
Group 9: *Antirrhinum*, *Beta*, *Cichorium*, *Taraxacum*	15.427***	55.703	4.072
Group 10: *Allium*, *Cynodon*, *Pseudotsuga*, *Stevia*, *Triphysaria*, *Zinnia*	42.779	49.258	2.600**
Group 11: *Asparagus*, *Fragaria*, *Zantedeschia*	130.585	45.719	2.757
Group 12: *Acorus*, *Ananas*, *Eucalyptus*, *Malus*	35.261	61.135	4.616
Group 13: *Ginkgo*, *Lycoris*, *Panax*, *Pennisetum*, *Picea*, *Zamia*	30.553	58.252	4.258
Group 14: *Aegilops*, *Oryza*, *Panicum*, *Saccharum*, *Secale*, *Sorghum*, *Zea*	31.365	60.619	6.723
Group 15: *Festuca*, *Hordeum*, *Lolium*, *Triticum*	46.006	63.169	6.435

A second measure of intergeneric similarity is mean rank difference in particular motif frequencies, which is presented respectively for zero, one, and two mismatches in Supplementary Tables 12, 13, and 14 (see Additional Files 12, 13, and 14). Here the null hypothesis is that the frequency of the given motif for the same number of genera was drawn at random from the entire set of 88 genera. The three tables present only those motifs with a 0.01 or lower probability of being ranked as they were by chance. Since only the 149 canonical motifs of two through five nucleotides were used, about 1.5 motifs are expected to be significant at p <= 0.01 for each group. With zero allowed mismatches, four of 18 natural groupings (*Malphigiales*, *Lamiales*, *Asparagales*, and "other monocots") did not exceed two significant motifs, versus two groups (*Malphigiales *and *Asparagales*) with one allowed mismatch and nine groups ("lower vascular plants", "other gymnosperms", *Caryophyllales*, *Rosaceae*, *Malphigiales*, *Lamiales*, *Asteraceae*, *Asparagales*, and "other monocots") with two allowed mismatches. There were fewest similarly ranked motifs within families and orders when two mismatches were allowed, and most when one mismatch was allowed. In part, rank consistency of a family or order depended on its values versus the mean for all 88 genera; families with very high or very low frequencies were more likely to exhibit a consistent ranking among the sampled genera. Since GC-rich motifs were especially abundant in *Poaceae*, the *Poaceae *had numerous GC-rich motifs that ranked more consistently than expected by chance. However, the *Solanaceae *ranked tightly for 5 to 16 motifs in spite of their low to middling frequencies. The *Asteraceae*, which like the *Solanaceae *exhibited a significantly low mean Euclidean distance among its genera, did not cohere as well in rankings, even though it had 52% of its rankings in the top or bottom 25% of the distribution versus 29% for the *Solanaceae*.

### Phylogenetic analysis of microsatellite frequencies

**Figure 3:**
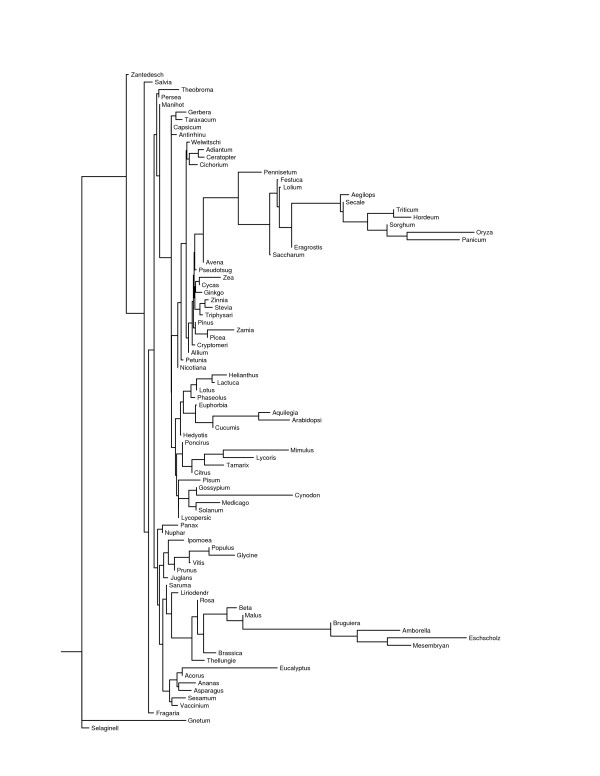
Maximum likelihood tree based on perfect microsatellites with motifs from two through five bases long. The names have been truncated to 10 letters.

**Figure 4:**
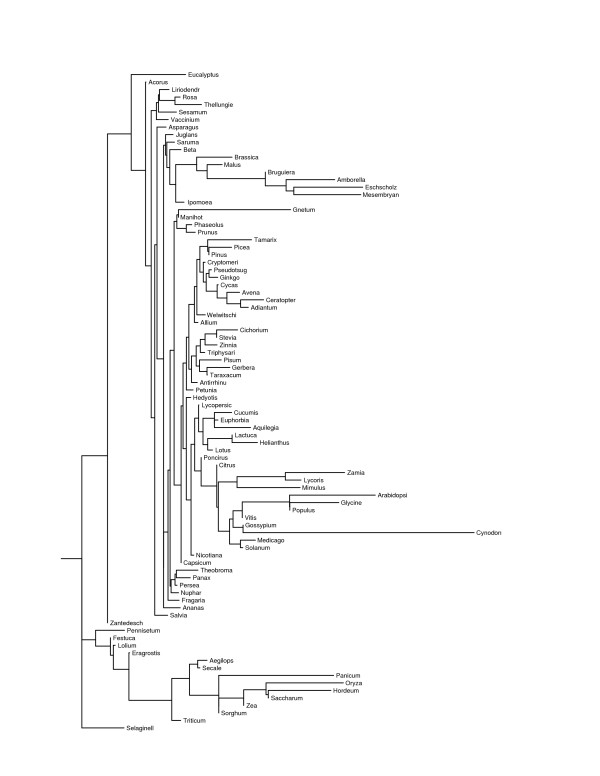
Maximum likelihood tree based on microsatellites with one allowed mismatch and motifs of two through five bases.

**Figure 5:**
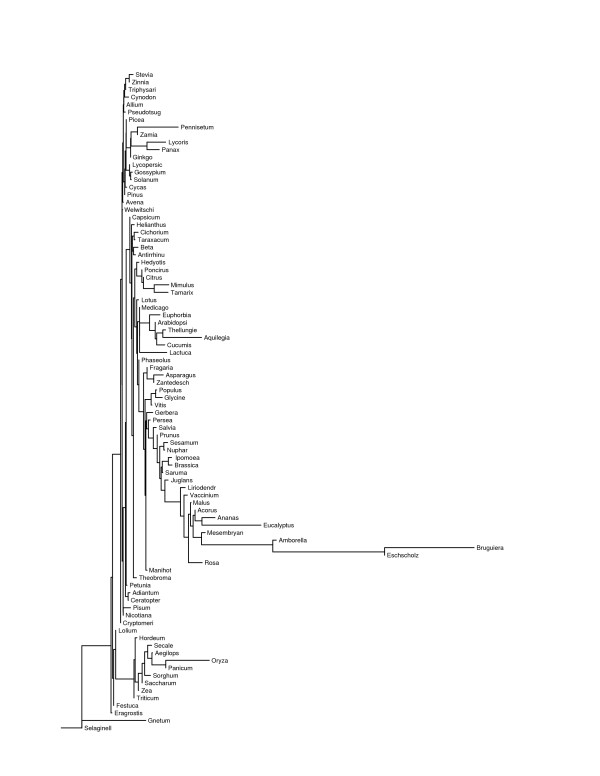
Maximum likelihood tree based on microsatellites with two allowed mismatches and motifs of two through five bases.

The program contml (continuous character maximum likelihood) in phylip 3.65 [25] used the 149 canonical two-base through five-base motif frequencies to construct three maximum-likelihood trees (Figs. 3, 4, and 5), one per level of allowed mismatches, that provide a convenient basis to consider intergeneric similarities. Each maximum-likelihood tree was the most likely tree to result from 300 trials with different input orders of the genera and global branch rearrangements of the initially formed tree. While it is not probable that a globally most likely tree was ever found, the greatest log-likelihoods were similar among runs within each number of allowed mismatches. However, the maximum found natural-log-likelihood differed greatly among the number of allowed mismatches (-25793.86 with none, -33852.89 with one, and -6671.66 with two), as did the mean Euclidean distance among all 88 genera (189.34 with none, 326.85 with one, and 55.18 with two). The mean Euclidean distances for total frequency within groups of related genera, as recognized in Tables 6 and 8, were significantly lower (p < 1e-06) when two mismatches were allowed. The longer motifs appeared to be more uniform among all vascular plants, and the recognized sequences were more random with two allowed mismatches. However, it is difficult to conclude from the likelihoods or mean distances that any one level of mismatches produced a more realistic phylogeny than did the others.

The three trees group together various, often quite unrelated, genera. Most of these groups are clades (terminal branches) within the tree, but others are merely close to some more basal node. The mean Euclidean distance among their members for dinucleotide through hexanucleotide motifs appears after the taxonomic groupings in Tables 6, 7, and 8. The mean rank differences among their members for dinucleotide through pentanucleotide motifs follow those of the taxonomic groupings in Supplementary Tables 12, 13, and 14. P-values are given for the null hypothesis that the observed ranking resulted from random sampling of the same number of genera from all 88 genera. Since there were 149 motifs involved, only those motifs that cohered at p <= 0.01 are presented. Supplementary Tables 12, 13, and 14 also include the mean ranking for each significant motif, from 1 for the lowest total frequency to 88 for the highest. Thus Supplementary Tables 12, 13, and 14 specify the motifs that most likely determined the placement of the group members in their respective maximum likelihood trees.

The most prominent clade in all three trees consisted of 11 to 13 out of 15 total genera of grasses (*Poaceae*), although *Cynodon *and one to three other genera fell outside of it in each case. Within the grass clade, members of the subfamilies *Pooideae *and *Panicoideae *freely interdigitated, although *Lolium *consistently associated with *Festuca*, and four genera of the pooid tribe *Triticeae *grouped together for perfect microsatellites. This clade exhibited increasing GC content distally, which is evident from the generally higher ranking of individual GC-rich motifs for the distal group 15 over group 16 in Supplementary Table 12, group 12 over group 13 in Supplementary Table 13, and group 14 over group 15 in Supplementary Table 14. This increased ranking was coordinated over many motifs and could reflect a general response to GC content of the transcriptome.

Another durable grouping included *Allium *and most of the gymnosperms (groups 2 and 3 in Supplementary Table 12, groups 1 and 2 in Supplementary Table 13), which ranked low for various motifs. The ferns *Adiantum *and *Ceratopteris *also ranked low for their most consistent motifs. *Glycine*, *Populus*, and *Vitis *grouped together in all three mismatch levels, with high rankings for AAAAT, AAAT, AAAC, and similar motifs with runs of A. *Citrus*, *Poncirus*, and *Mimulus *also clustered together at each mismatch level; *Tamarix *associated with these three with zero or two allowed mismatches, and *Lycoris *associated with these three with zero or one mismatch. *Acorus*, *Ananas*, and *Eucalyptus *associated for zero or two mismatches, but went to separate branches with one mismatch. *Thellungiella *grouped with *Brassica *with zero mismatches, *Arabidopsis *with two mismatches, and neither with one mismatch. The main message of Supplementary Tables 12, 13, and 14, is that there are few prominent groups that remain consistent over numbers of mismatches or minimum locus length. In particular, the *Solanaceae*, which cluster close together in terms of Euclidean distance, did not cluster in any of the trees, except that *Solanum *grouped with *Lycopersicon *with zero or two allowed mismatches. Similarly, the *Asteraceae *resided in four groups for perfect microsatellites, two groups for one allowed mismatch, and five groups for two allowed mismatches, even though their genera were significantly close together by Euclidean distance. Furthermore, there was no particular correlation of GC-content or GC-rich motif frequencies to higher taxa. Apart from the *Poaceae*, the microsatellites of monocots were not particularly GC-rich, and one monocot (*Lycoris*) had the second most AT-rich microsatellites of all 88 genera for zero or one allowed mismatch, even though its EST collection ranked 21^st ^in AT content for zero mismatches.

### Microsatellite frequency in relation to genome size

The total per-megabase frequency of perfect microsatellites was inversely correlated with genome size. This was evident anecdotally from the lowest ranking genera in microsatellite frequency (Table 9), all of which (where known) exceeded the median 2c value of 2.56 pg for EST sequence donors in the 78 genera where DNA content was available [26-28]. The negative correlation was also evident by linear regression of microsatellite frequency against 1c DNA content over these same genera. When all 78 genera were considered together, the regression slope was -21.77 loci per picogram of 1c DNA content (p = 0.036), whereas without the *Poaceae *the slope was -20.43 (p = 0.008). The correlation was not significant within the *Poaceae*, although the slope was still negative (-10.71, p = 0.90), because *Zea *has a smaller genome and a lower microsatellite frequency than the pooid genera *Triticum*, *Hordeum*, *Secale*, *Avena*, *Festuca*, and *Lolium*. Nevertheless, *Zea *has arguably the largest monoploid genome in the panicoid grasses, and it has likely expanded recently during its evolution, apart from its doubled chromosome number [29]. However, this negative correlation almost disappeared when gymnosperms were excluded from the sample (slope = -6.44, p = 0.69), indicating a more direct relationship to phylogeny than to genome size per se.

**Table 9:** DNA content of genera with lowest total microsatellite frequencies

Genus	Total frequency/MB	2c DNA content (pg)	Reference
*Zea*	647.59	5.45	[28]
*Adiantum*	676.63	9.30	[27]
*Cycas*	741.21	25.50	[26]
*Cichorium*	804.71	Not available	
*Welwitschia*	805.14	14.40	[26]
*Ginkgo*	822.56	19.90	[26]
*Cryptomeria*	844.84	22.09	[26]
*Triphysaria*	848.09	Not available	
*Allium*	851.01	33.50	[28]
*Pseudotsuga*	853.76	38.10	[26]

When total perfect microsatellite frequencies were itemized by motif length and regressed versus 1c DNA content over all 78 available genera, the resulting slopes were significantly negative for dinucleotide (-5.04, p = 0.00096) and trinucleotide (-7.62, p = 0.0064) motifs, but not for tetranucleotide motifs (-2.88, p = 0.42). Within the *Poaceae*, the slopes were negative but not significantly so (p > 0.30), and without the *Poaceae*, the slopes became even more significant for di- and trinucleotide motifs, and remained nonsignificant (p = 0.37) for tetranucleotide motifs. Finally, without the gymnosperms, the slopes were negative but not significant for all three motif lengths.

To test the possibility that the transcriptomes of these large-genomed genera are being diluted by transcription of transposable elements, which might be more abundant in large genomes, the phrap-generated singlets and contigs of *Allium*, *Picea*, *Pinus*, *Arabidopsis*, *Glycine*, and *Oryza*, were subjected to tblastx versus RepBase 3.11 at a cut-off e-value of 1e-10. The respective 2c values of these genera are 33.5, 40.4, 44.2, 0.32, 1.14, and 1.00 pg [26,28], when tetraploidy is accounted for in *Glycine*. The percentage of sequences matching repetitive elements was 16.9 in *Oryza*, 3.9 in *Allium *and *Glycine*, 3.5 in *Pinus*, 2.8 in *Picea*, and 2.7 in *Arabidopsis*, which did not fit the expectation that the three large genomes would have the three highest percentages.

### Sources of long motifs

There are three interesting sources of repeated long motifs: repeated domains in proteins, tandem repeats in transcribed repetitive elements such as retrotransposons, and exon repetition resulting from mRNA editing. Several tactics were tried to quantify these sources. The first was to tblastx the 18374 repeated motifs of 30 or more nucleotides, derived from all 88 genera, against RepBase 3.11. These hits were frequent (0.298 of the sequences hit at an e-value less than 1, and the mean was 7.57 matched amino acids per hit) but unimpressive, mostly with e-values greater than 0.01. This was because the motifs were generally short and incompletely matched, representing a nonconservative subsequence in the repetitive element. This led to the second tactic, Monte Carlo simulation of sequences that matched the real repeated motifs in length, followed by tblastx against RepBase. The simulation took independent account of codon usage in each genus (which will be presented elsewhere), and 0.252 of the simulated motifs hit at an e-value less than 1, with a mean of 7.85 matched amino acids per hit. Although these are close to the observed values for the real motifs, a one-sided, paired-data t-test gave t = 4.742 for hits per query and t = -2.952 for identical amino acids per hit, both of which were significant at p = 0.002 with 261 degrees of freedom (88 genera, 3 allowed mismatches, and two combinations that gave no long motifs). Thus there were significantly more hits in the real data, but the hits were shorter than in the simulation, and hits to random sequence could account for about 85 percent of the observed frequency.

The third tactic was tblastx of the whole sequences that contained the long motifs against RepBase. Because of the longer query sequences, the cutoff e-value was decreased to 0.01, but still 8850 sequences hit out of 18374 distinct long-motif-bearing sequences. Furthermore, there were a few close matches to repetitive elements: 3211 (0.175 of the total) hit at 1e-10, 644 (0.035 of the total) hit at 1e-20, 141 (0.008) hit at 1e-30, 38 (0.002) hit at 1e-60, and the closest hit was to a CINFUL2A_I element at 1e-144. At an e-value of 1e-10, the most frequently hit elements were ENSPM4_OS (1012 hits out of 3211 total), ENSPM7_OS (198 hits), MERMITE18D (633 hits), TREP60 (424 hits), SZ-6IN (225 hits), MUDRN3_OS (67 hits), XILON1_ZM_LTR (64 hits), and CACTA-L (59 hits). There were 20 hits to CINFUL2A_I at 1e-10, and 14 of them were closer than 1e-60. Other slowly evolving elements that were hit at 1e-60 or less included HUCK1-LTR_ZM (7 hits) and ZEON1_ZM (3 hits). Thus tblastx of the whole sequences also suggests that 15 to 20% of the long motifs are direct repeats within transcribed repetitive elements.

**Figure 6:**
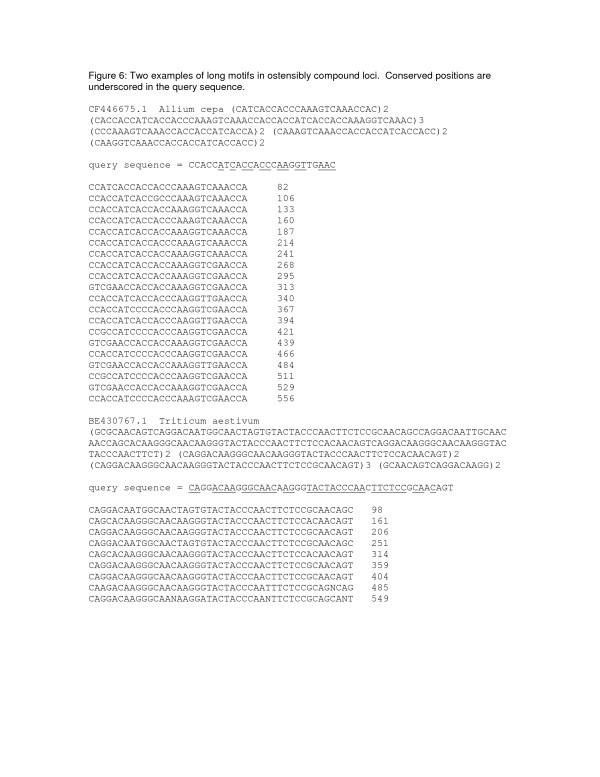
Two examples of imperfect minisatellite loci.

High copy numbers of long motifs were particularly well represented in *Populus*, *Pinus*, *Lycopersicon*, *Solanum*, and *Helianthus*. Many of the longest repeats were reported as parts of compound microsatellites having several submotifs of equal length. Two examples of such loci are presented in Fig. 6. Both examples were imperfect simple repeats of a long motif, with more mismatches over repeats than were allowed in this study. The appearance of a compound locus resulted from the interruption of the locus by more than two mismatches, often resulting in the same repeated subsequence being recognized in more than one frame at different positions within the locus.

With blastx unfiltered for low-complexity sequence (-F F), the first sequence in Fig. 6 (CF446675.1 from *Allium cepa*) hits a number of cell-wall or membrane proteins from vascular plants and algae. In containing domains of proline, serine, and lysine, it is similar to CAA84230, an extensin from maize pollen, although the closest matches (1e-61) are to algal pherophorin proteins. The second sequence, BE430767.1 from *Triticum aestivum*, is a high-molecular-weight glutenin; the closest blastx hits are around 1e-80. In a blastx search of all 77 sequences from all 88 genera with at least six copies of a motif of at least 18 nucleotides, 63 sequences hit proteins in GenBank's nr database at e-values less than 1e-08. However, 30 of the hits were to the same entry, XP_344805.1, a putative protein from rat. These 30 sequences, which contained a 23-mer that was repeated up to 31 times in *Populus euphratica *(GenBank accession AJ770915.1), came from *P. euphratica*, *Populus tremula *x *Populus tremuloides*, *Lycopersicon esculentum*, *Helianthus argophyllus*, and *Pinus taeda*. The persistent redundancy suggests that the sequences represent a small multigene family that is widely distributed in angiosperms and gymnosperms.

## Discussion

The 77% reduction of total sequence length from redundant to nonredundant datasets in this study compares to an 85% reduction in number of ESTs from redundant to nonredundant datasets in [3]. Since the current study attempted to conserve deviant repeat loci as much as possible, it is likely that limited redundancy remains in the repeat loci reported here. This residual redundancy could distort the repeat frequencies somewhat from their values in the coding genomic sequence. There is a conflict between the goals of mining every microsatellite from an EST collection, and accurately extracting the motif frequencies from the coding sequence.

The slowest step of the pipeline is phrap, followed by the initial repeat finder. The run time of the phrap step is strongly influenced by the amount and pattern of redundancy within the dataset. The run time of the repeat finder increases quadratically with the number of motif lengths sought and linearly with the length of examined sequence; this time is small for motifs of less than 10 nucleotides. Although the repeat finder's algorithm is not particularly efficient, it is effective, and larger datasets can be divided and run in parallel.

The decision to allow the independent reporting of short and long repeats from the same subsequence, so that embedded classical microsatellites would not be overlooked in longer repetitive sequences, inflated the reported count of motifs that are part of very long motifs, probably by several percent.

The necessity to repeat phrap on large datasets probably merges distinct but similar sequences excessively, leading to a loss of microsatellites but also a canceling loss of "nonredundant" sequence to host them. The effect is not consistent, but it might be responsible for the low reported microsatellite frequencies in *Zea*.

Low-quality sequence was not completely excluded from the analysis. Particularly bothersome are relatively short tracts of low-quality sequence within otherwise accurate sequence, since these can appear as tracts of a single nucleotide and inflate the count of microsatellite motifs like AAAC, AAAG, and AAAT. Any rules-based removal of such subsequences would likely also remove legitimate instances of motifs that contain runs of a single nucleotide.

The observed satellite frequencies exceed those expected by chance in random sequence at every motif length and mismatch level, but the difference was least (and probably not significant) for eight- through 11-base motifs with one or two allowed mismatches. A more realistic model of random codon order, taking into account the observed codon preference of the sequences, probably would yield higher expected repeat frequencies. Such a model could provide testable predictions of relative motif frequencies to be expected in random sequence, that could be compared to the ranking of observed frequencies.

The low microsatellite frequency in *Allium *and conifers, which have very large diploid genomes with 2c > 20 pg, runs counter to the intuitive expectation that large genomes should have more and longer microsatellites [30]. However, studies in a grasshopper [30], fungi [31], and vertebrates [32] have demonstrated that the frequency and length of genomic microsatellite loci are not correlated to 2c DNA content. The loss of significantly negative correlation upon excluding gymnosperms accords with a relationship to phylogeny more than to genome size in itself, and thus plants probably resemble animals in their relationship of microsatellite frequency to genome size. An open question is how much the transcriptome is diluted by the individually infrequent transcription of a multitude of microsatellite-poor repetitive elements in large genomes. The tblastx results for six genera here indicate no correlation of transposable-element transcription to genome size. Instead, the transposable elements of *Oryza *might only be better known and more represented in RepBase than the rest, even though full genomic sequence is available for both *Oryza *and *Arabidopsis*.

Polymorphism is as frequent for pentanucleotide and hexanucleotide motifs as it is for the classical di- and trinucleotide microsatellites, although the repeat counts are lower. Such loci ought to be considered as potential genetic markers.

The pipeline offers primers to amplify most of the detected repeats, but the success of amplification depends upon the nonredundancy of the primer sequences in the genome, the distribution of primers among exons, and the success of phrap in reconstructing consensus EST sequences. The EST sequences provide little information about primer redundancy in the genome, since they do not include nontranscribed sequence, which comprises most of the genome, and since many genes belong to multigene families of varying size and complexity. The mature sequences also do not provide the locations of splice sites, and amplification will fail if a suggested primer straddles a splice site or if suggested primers are separated by too large an intron. Finally, there is always a chance that phrap has misassembled similar sequences, such that a primer pair suggested from a contig has no identical spacing or even identity in the genome.

Contamination of the EST databases with ribosomal RNA seems to be minimal, or the length of individual rRNA genes exceeds the 250-base cutoff used in this study. The amount of contamination with untranscribed genomic sequence remains unknown in these collections.

Microsatellite sequences often evolve faster than the taxa that bear them, although closest relatives usually retain similar microsatellite frequencies. This holds not only for frequency but also for polymorphism and especially mean repeat count, and it is consistent with the limited transferability of microsatellites among less related species. Repeated subsequences are probably subject to natural selection, like almost anything else that is transcribed.

Several other studies have reported motif frequencies from individual genera that were analyzed here. Supplementary Table 15 (see Additional File 15) presents frequencies of di- and trinucleotide motifs in perfect, simple microsatellite loci reported elsewhere from 16 genera, along with the minimum lengths of the loci, for comparison with the results reported here. A few reports provided only the most abundant motifs, or reported the same canonical motif as two or more variants that were summed for the table. Wherever all four dinucleotide motifs were given, the reported frequency ranking matched that observed here, except in *Citrus*, where the minimum dinucleotide locus was only 12 nucleotides. The relative frequencies of trinucleotide motifs differed more among genera and between studies, because of differences in sample size and the handling of EST redundancy. This affects mostly the lower-ranking motifs. In some cases, not all of the possible motifs were reported, and possibly they had not been searched for. It is not clear what happened with *Pinus*, which does not accord with the current results or with the frequencies in the related genus *Picea*. Overall, the dinucleotide motifs rank as AG, AT, AC, and CG, but conifers, *Gossypium*, and *Ipomoea *rank AT, AG, AC, and CG. The present study more consistently ranks AAT and ACT last or close to last than do the cited reports, and it uniformly ranks CCG first in the *Poaceae*.

Kantety et al. [3] also reported frequencies by motif length in *Triticum*, *Hordeum*, *Oryza*, *Sorghum*, and *Zea*, using minimum locus lengths of 18 nucleotides for dinucleotide and trinucleotide motifs and 20 nucleotides for tetranucleotide motifs. Motifs of two, three, or four nucleotides respectively represented 19, 72, and 9 percent of the total, in modest contrast to 15, 70, and 15 percent here.

Cardle et al. [17] used respective minimum lengths of 15, 16, and 20 nucleotides for mononucleotide, tetranucleotide, and pentanucleotide motifs in *Arabidopsis*. They found A to exceed C by about 10 to 1, versus an excess of only 2:1 here, which probably reflects the strategy used here to eliminate polyA tails from the EST collections. They found very few tetranucleotide motifs in ESTs; AAAC was the most common. Here, tetranucleotide motifs were about 30% as common as dinucleotide motifs. AAAG was nearly twice as common as AAAC, and the next ranked motifs were ACAT, AAAT, AGAT, AATG, and ACCC, with 21 of 33 canonical motifs represented at least once. They found no pentanucleotide motifs in the EST sequences, whereas here such motifs were 20% as common as trinucleotide motifs and represented 66 of the 102 possible canonical motifs, both because of the much larger sample size and the shorter minimum microsatellite locus. In marked contrast to the present study, they found very few compound microsatellite loci, probably because they used the full-length criteria for all members of compound loci.

Han et al. [33] used minimum locus lengths of 10, 12, 16, 20, and 24 nucleotides respectively for motifs of two, three, four, five, and six bases in *Gossypium *ESTs. They reported respective relative frequencies of 30, 59.1, 6.4, 1.8, and 2.7%, versus 14.6, 35.6, 7.4, 17.8, and 14.4% here, where the minimum locus length was shorter for the longer motifs.

From 15 to 20% of the longer instances represent repeated subsequence in repetitive elements, such as transposons. Most or all of the rest can be attributed to repeated subsequences within genes, such as those that encode cell-wall or seed-storage proteins. O'Dushlaine et al. [34] found that about six percent of human proteins manifest tandem-repeat polymorphisms. The repeated subsequences do not necessarily preserve reading frame, although there is a substantial excess of those that do. Repeats that disrupt reading frame have been invoked in the evolution of nine types of unstructured proteins in animals [35], including the C-terminal domain of eukaryotic RNA polymerase II. O'Dushlaine et al. [34] suggest that up to one percent of human protein unigenes contain frame-shifting repeats, and that such proteins tend to be involved in protein-protein interactions. The present study could not substantiate any examples of exon repetition arising upon RNA splicing, even though specific instances of exon repetition and exon scrambling have been demonstrated to result from intermolecular RNA splicing in human beings [36] and five other animal species [37]. Also, the mean GC content is higher for motifs that retain reading frame, and this accords with a higher GC content within coding sequence than elsewhere.

Inukai [38] investigated minisatellite loci in 5.3 megabases of genomic sequence in *Oryza sativa*. The loci had at least three copies of a motif at least 10 nucleotides long. Based on blast searches within the sampled genomic sequence, 183 loci were considered multilocus, i.e., occurring within similar sequence at dispersed positions in the genome, and 37 were single-locus. The loci were ascribed to transposable elements if their flanking sequence met criteria for long-terminal repeats, inverted repeats, or target-site duplication, or if they matched database entries for rice transposable elements. Of the 41 single-locus minisatellites, eight (19.5%) were embedded in transposable elements, comparable to the 15 to 20% of minisatellites estimated to belong to repetitive elements in the current study. While all 183 of Inukai's multilocus minisatellites belonged to transposable elements, it is unlikely that these elements were transcribed at a significant frequency, so one would not expect them to dominate the minisatellites within the transcriptome. The En/Spm family of class II transposable elements was one of the most common sources of minisatellites in Inukai's study, and it was the leading repetitive-element source of minisatellites in the current study. Also, minisatellite GC content in his study behaved the same as in the current study, exceeding the GC content of the flanking sequence when that GC content was high, and falling short when that GC content was low.

Tissue specificity is a generally underestimated source of microsatellite frequency variation in EST collections. Bilgen et al. [39] demonstrated this for ESTs drawn from 11 different tissues or developmental stages in *Arabidopsis thaliana*, where the relative frequency of the leading dinucleotide motif, AG, varied from 31.6% in seedlings to 55.5% in developing seeds. The current study did not try to select subsets of EST collections on the basis of annotation, so it cannot comment on the degree to which the phylogenetic analysis of motif frequencies was perturbed by tissue representation in the collections. It is potentially quite significant. Also, transcriptional slippage is a potential source of spurious microsatellite alleles in cDNA libraries [34], but the measured frequency of such slippage is only about 0.02% of all mRNA molecules in ubiquitin-B and amyloid precursor protein in human [40]. Therefore, transcription slippage has likely occurred at some very low frequency in the founding mRNA of the EST datasets examined here.

Finally, microsatellites stand in the eye of the beholder, i.e., the threshold criteria for patterned sequence are rather arbitrary and there is a continuum from perfect microsatellites to random sequence. Although a minimum locus length of 15 nucleotides is appropriate for short microsatellite motifs, a minimum of 20 or more is less likely to pick up randomly occurring doublings of longer motifs. Between 10 and 20% of patterned-sequence loci are polymorphic within EST collections for motif lengths from two to six nucleotides, suggesting that the longer motifs should also be sought out and used wherever di- and tri-nucleotide motifs are used now. Frequently the polymorphism results from indels in the flanking sequence, in addition to or instead of variation in repeat count in the microsatellite itself. Patterned sequence appears to be subject to natural selection and to evolve more rapidly than the transcriptome at large.

## Methods

### Data source

DNA sequences were extracted from gbest flat files in GenBank release 150, dated 15 October 2005 [41]. For each accession, the genus was recognized as the third word (tract of non-whitespace text) in the line that contained the string "ORGANISM". Sequences were counted by genus, and sequences were written to separate flat files for all 88 genera that were represented by at least 3000 sequences.

### Removal of vector and low-quality sequence

Each genus was processed independently. Its sequence file was first searched for vector sequences by blastn [42] against UniVec [43]. A Perl 5.8 script then removed the vector-matching subsequences, except for the telomeric repeats of YAC vectors. A second Perl script trimmed off tracts of at least 10 consecutive A's or T's within the first 60 nucleotides from either end of the vector-trimmed sequences, plus any sequence distal to these tracts.

### Recognition and processing of repeated motifs

A generic repeat finder, written in C, searched for repeated motifs from 1 to 250 nucleotides long in the trimmed sequences, without regard for redundancy in the motif. A Perl script then recognized motifs that were multiples of shorter motifs, and purged the list of repeats to retain only the shorter motifs. This script allowed the recognition of overlapping motifs provided that one was sufficiently longer than the other, and was not a multiple of it. In this way, classical microsatellites were sometimes recognized within longer minisatellites. Another Perl script then identified all instances where different blocks of repeats occurred within five nucleotides of one another, thereby comprising a compound locus. From this script emerged the list of all simple and compound sites of patterned sequence within the set of ESTs. To eliminate duplicate and nearly duplicate sequences, phrap [21] was applied to only the sequences that contained patterned sequence. Phrap produced a list of singlets and a list of contigs. The contigs were parsed for the longest contributing sequence that contained patterned sequence at a particular position in the contig, where position was a window of nine nucleotides centered at the midpoint of a patterned sequence. Therefore, primers were always chosen from a real sequence, even if the contig was misassembled. The singlets and chosen contig-contributors were output as a list of motifs, positions, and host-sequence identities. This list, and the sequences that it specified, were reformatted for input to primer3 [22], which returned wherever possible six pairs of primers per locus. The primers were chosen to position the microsatellite locus off center in the expected amplification product, closest to a specifiable ratio of short-side length to long-side length. This improved the chances of successful verification on agarose gels when a motif-specific third primer is included. Finally, the chosen primers were compared to all the sequences in the nonredundant set resulting from phrap, counting matches to indicate the likely frequency of the amplified sequence in the genome.

The phrap program could not successfully process large datasets, even under its manyreads option, because it exhausted system memory. In these cases, the vector-polyA-trimmed input file was split up so that no piece had more than about 150000 sequences, and the entire pipeline ran independently on the pieces. A Perl script then identified the contributing sequences for each piece, and the pipeline was rerun on only these contributing sequences. For the largest datasets, e.g., *Triticum *and *Zea*, it was necessary to undertake this strategy twice, so as to maintain datasets of manageable size throughout the processing.

### Statistical analysis

Other Perl scripts extracted canonical motifs from the pipeline's output and tabulated repeat counts, GC content, frequency of polymorphism, and so on. The canonical form of a motif was the lexicographically least form obtained upon all possible rotations of the motif and its reverse complement. Thus TCA reduced to ATC, and TC reduced to AG. The results were grouped by canonical motif, even though the different contributing motifs might have independent biological functions that depend upon reading frame. Statistical significance was tested by empirical distributions of 20000 to 50000 randomly drawn samples of the same size, wherever a parametric distribution was unavailable, as in the case of mean Euclidean distances among genera or mean motif rankings.

Microsatellite frequency per megabase was estimated by dividing counts of post-phrap loci by numbers of nucleotides in non-redundant trimmed sequences, which were obtained by using phrap independently on all the trimmed sequences. As above, the larger datasets required division and reprocessing of subsets through phrap.

### Repeat frequency in random sequence

The observed microsatellite frequency was compared to that expected by chance in random sequences of the same length as the observed sequences. If the bases occur at equal frequency (0.25), the expected frequency of tandem repeats of length m with k mismatching nucleotides in a sequence of length n is:

F = (n - 2m +1) * 4^-m ^* 3^k ^* m!/(k! * (m - k)!)

The sum of F for k = 0, 1, and 2 gives the expected frequency of tandem repeats given two allowed mismatches, and the sum of such sums over all sequence lengths in the EST collection gives the expected frequency of repeats of motif length m. Similarly, the expected frequency of triple tandem repeats is

F = (n - 3m + 1) * 4^-2m ^* 3^k ^* (2m)!/(k! * (2m - k)!),

whose sum for k = 0, 1, and 2 is approximately the expected frequency of triple tandem repeats with two allowed mismatches. The sum is a bit in excess because two mismatches in the second repeat disqualify the repeat block as a triple tandem repeat. The comparison for double tandem repeats was done for motif lengths where the minimum locus contained two repeats, i.e., where the motif was at least eight nucleotides long. Below that, the triple tandem repeats formula was used for motifs from five through seven nucleotides long. The expected ratio of double to triple repeats is approximately 4^m^.

### Detection of repetitive elements

To test the possibility that repeated motifs were part of repetitive sequences, particularly retrotransposons that were being transcribed at individually low but collectively substantial frequency, motifs of at least 30 bases were subjected to tblastx [42] against RepBase 3.11 [23]. Also subjected to tblastx against RepBase was a parallel set of synthetic sequences, constructed at random to the same lengths as the real motifs in accordance with codon usage in each genus. This synthetic set relied on random deviates uniformly distributed in [0,1), produced by the Mersenne Twister generator [44] as function runif() in R [24]. This set allowed an appraisal of the biological significance of hits of the real repeated motifs to various repetitive elements. Also, the real sequences that contained 30-base or longer repeated motifs were subjected to tblastx against RepBase, to identify long but diffuse hits that might have arisen from the rapid evolution of repetitive elements.

## Abbreviations

compfreq: frequency of compound (multi-motif) microsatellites containing this motif per megabase of EST sequence, after trimming of vector and poly-A tails

comppoly: relative frequency of polymorphic to total compound microsatellites that contain this motif

comprepe: mean repeat count of this motif in compound microsatellites that contain this motif

fracsimp: relative frequency of simple microsatellites among total microsatellites that contain this motif

simpfreq: frequency of simple microsatellites of this motif per megabase of trimmed EST sequence

simppoly: relative frequency of polymorphic to total simple microsatellites based on this motif

simprepe: mean repeat count of this motif in simple microsatellites based on this motif

totfreq: total frequency of simple and compound microsatellites that contain this motif per megabase of trimmed EST sequence

## Supplementary Material

Additional File 1 Observed and expected per-megabase frequencies by motif length in a subset of 12 genera. This Excel table lists total per-megabase frequencies of repeated motifs, grouped by motif length, in 12 of the 88 total genera. For comparison, it also lists the frequencies expected by chance under Equations 1 and 2 for random sequences matched in length to the observed sequences. Click here for file

Additional File 2 Per-megabase frequency of canonical motifs in perfect microsatellites in 88 genera of vascular plants. This Excel table lists numerical values of the eight quantities defined in the Abbreviations for all canonical motifs from one to six bases long observed in perfect microsatellites in the 88 genera examined. Canonical motifs are motifs in their lexicographically least form, considering all possible rotations and reverse complementation. For example, AC is the canonical form of AC, CA, GT, and TG. Click here for file

Additional File 3 Per-megabase frequency of canonical motifs in microsatellites in 88 genera, allowing one mismatched repeat position. This Excel table parallels Additional file 2. Here the values of the eight quantities defined in the Abbreviations are tabulated for canonical motifs in microsatellites with up to one mismatch allowed among repeats. Click here for file

Additional File 4 Per-megabase frequency of canonical motifs in microsatellites in 88 genera, allowing two mismatched repeat positions. This Excel table is also parallels Additional file 2, with up to two mismatches allowed among repeats. Click here for file

Additional File 5 Collective per-megabase frequency, repeat count, and polymorphism by canonical microsatellite motif in perfect loci. This Excel table lists values, sorted from greatest to least, for the eight quantities defined in the Abbreviations, collected over all 88 genera. It thus provides the overall ranking of motifs for these quantities. Click here for file

Additional File 6 Collective per-megabase frequency, repeat count, and polymorphism by canonical microsatellite motif with one allowed mismatched repeat position. This Excel table parallels Additional File 5, allowing up to one mismatched nucleotide among motifs in the microsatellite locus. Click here for file

Additional File 7 Collective per-megabase frequency, repeat count, and polymorphism by canonical microsatellite motif with two allowed mismatched repeat positions. This Excel table also parallels Additional File 5, allowing up to two mismatches among motifs in the microsatellite locus. Click here for file

Additional File 8 Motif rankings by number of genera in which motif held first place for frequency, repeat count, and polymorphism in perfect microsatellites. For the eight quantities defined in the Abbreviations, this Excel table lists the number of genera in which each motif ranked first. Click here for file

Additional File 9 Motif rankings by number of genera in which the motif held first place for frequency, repeat count, and polymorphism in microsatellites with one allowed mismatched repeat position. This Excel table parallels Additional File 8, allowing up to one mismatched nucleotide among motifs in the microsatellite locus. Click here for file

Additional File 10 Motif rankings by number of genera in which motif held first place for frequency, repeat count, and polymorphism in microsatellites with two allowed mismatched repeat positions. This Excel table parallels Additional File 9, allowing up to two mismatched nucleotides among motifs in the microsatellite locus. Click here for file

Additional File 11 Regression coefficients of microsatellite characteristics versus GC content. For the eight properties defined in the Abbreviations, this Excel table provides regression coefficients to a quadratic model of property versus motif GC content. Significance is also indicated. Click here for file

Additional File 12 Mean within-group ranking difference of genera for total motif frequency in perfect microsatellite loci. For groups of genera defined by the maximum-likelihood tree of perfect-microsatellite similarity in Fig. 3, this Excel table lists the motifs that were the most consistently ranked and therefore that most likely defined the groups. The highest frequency has the highest ranking. Click here for file

Additional File 13 Mean within-group ranking difference of genera for total motif frequency in microsatellite loci with one allowed mismatch. This Excel table parallels Additional File 12, but the groups were drawn from Fig. 4, where one nucleotide mismatch was allowed. Click here for file

Additional File 14 Mean within-group ranking difference of genera for total motif frequency in microsatellite loci with two allowed mismatches. This Excel table parallels Additional File 12, but the groups were drawn from Fig. 5, where two mismatched nucleotides were allowed. Click here for file

Additional File 15 Observed and published microsatellite motif frequency rankings in 16 genera. This Excel table lists observed and previously published rankings of di- and tri-nucleotide motif frequencies in these genera, and provides the size of the minimum microsatellite locus recognized in each case. Click here for file
